# Parboiled Rice and Glycemic Control: Effects on Postprandial Glucose, Insulin Sensitivity, and Incretin Response in Healthy and Type 2 Diabetic Individuals, a Pilot Study

**DOI:** 10.3390/foods14111905

**Published:** 2025-05-27

**Authors:** Sara Alkandari, Tasleem A. Zafar, Suleiman Al-Sabah, Mohammed Abu Farha, Jehad Abubaker, Fahd Al-Mulla

**Affiliations:** 1 Ahmadi Hospital, Kuwait Oil Company (KOC), Al-Ahmadi 61008, Kuwait; sakandari@kockw.com; 2 Department of Food Science and Nutrition, College of Life Sciences, Kuwait University, Shadadiya 12037, Kuwait; 3 Department of Pharmacology & Toxicology, Faculty of Medicine, Kuwait University, Kuwait City 13060, Kuwait; sulaiman.alsabah@ku.edu.kw; 4 Department of Biochemistry and Molecular Biology, Dasman Diabetes Institute, Kuwait City 15462, Kuwait; mohamed.abufarha@dasmaninstitute.org (M.A.F.); jehad.abubakr@dasmaninstitute.org (J.A.); 5 Department of Genetics and Bioinformatics, Dasman Diabetes Institute, Kuwait City 15462, Kuwait; fahd.almulla@dasmaninstitute.org

**Keywords:** type 2 diabetes mellitus, healthy subjects, insulin resistance, insulin sensitivity, Glp-1, parboiled rice, white rice

## Abstract

Type 2 diabetes mellitus (T2DM) represents a significant global health burden, especially in populations where rice constitutes a dietary staple. Parboiled rice (PBR), known for its lower glycemic index compared to conventional white rice (WR), may offer benefits in managing postprandial hyperglycemia. Nevertheless, the impact of PBR consumption on insulin sensitivity, β-cell function, and incretin hormone responses remains poorly understood. Methods: This randomized crossover pilot study aimed to assess and compare the acute effects of PBR and WR intake on postprandial glucose regulation, insulin sensitivity, β-cell functionality, and glucagon-like peptide-1 (GLP-1) responses in healthy subjects and individuals with T2DM. A total of 20 participants were recruited and evenly allocated into healthy (*n* = 10) and T2DM (*n* = 10) groups. Following the ingestion of either PBR or WR, blood samples were collected at fasting and various postprandial intervals to determine glucose, insulin, and GLP-1 levels. Insulin sensitivity and β-cell function were evaluated using HOMA-IR, Matsuda Index (MI), and Disposition Index (DI). Results: As expected, T2DM participants exhibited significantly elevated fasting glucose and insulin levels compared to healthy controls. Consumption of PBR led to significantly lower postprandial glucose responses in healthy subjects relative to WR. Although a similar trend of reduced glucose levels was observed in T2DM subjects after PBR intake, this reduction did not reach statistical significance. Parallel trends were observed in insulin secretion patterns. Moreover, GLP-1 responses were notably diminished in T2DM individuals compared to healthy participants. Importantly, MI and DI values significantly increased after PBR consumption in healthy individuals compared to those with T2DM, indicating improved insulin sensitivity and β-cell responsiveness. Conclusions: These preliminary findings suggest that PBR consumption may confer beneficial effects by lowering postprandial glucose and enhancing insulin sensitivity. Further studies with larger cohorts are warranted to confirm these outcomes and elucidate the physiological mechanisms behind PBR’s potential role in dietary management strategies for T2DM.

## 1. Introduction

Insulin resistance is a hallmark of Type 2 Diabetes Mellitus (T2DM), a debilitating metabolic disorder that is rapidly increasing worldwide, particularly in developing countries. In Kuwait, the prevalence of T2D is 23.1%, the second highest globally after Saudi Arabia (23.9%) [1]. According to the American Diabetes Association (ADA), maintaining blood glucose levels within the normal range is a key goal in diabetes management. This is primarily achieved through lifestyle modifications, including diet and medication [2,3]. Also, frequent elevated blood glucose excursions in healthy individuals lead to increased HbA1c level, which is a known risk factor for developing type 2 diabetes [4].

Blood glucose regulation relies on a dynamic balance between insulin secretion from pancreatic β-cells and glucose uptake by peripheral tissues. Hyperglycemia can result from either reduced insulin secretion due to β-cell dysfunction or insulin resistance (IR), which impairs glucose uptake [5]. If left uncontrolled, chronic hyperglycemia can lead to macrovascular and microvascular complications, including retinopathy, nephropathy, and neuropathy [6]. Despite receiving hypoglycemic treatment, many individuals with T2DM struggle to maintain normal blood glucose levels. This may be due to the inability of these medications to specifically target either insulin resistance or β-cell dysfunction.

Insulin resistance is commonly assessed using the Homeostasis Model of Assessment for Insulin Resistance (HOMA-IR), calculated from fasting glucose and insulin levels [7]. While HOMA-IR is widely used, it primarily reflects hepatic insulin resistance and may underestimate IR at the peripheral tissue level [8]. In contrast, β-cell function is assessed using HOMA-B, which evaluates the pancreatic β-cells’ ability to compensate for IR [9]. Another key metric for β-cell function is the disposition index (DI), which reflects both insulin secretion and glucose uptake efficiency [10]. A lower DI indicates reduced β-cell function and an inability to compensate for IR [11]. The Matsuda Index (MI) is a tool that measures insulin sensitivity, incorporating both fasting and postprandial glucose and insulin levels following an oral glucose challenge [12]. Unlike HOMA-IR, MI provides a more comprehensive measure of whole-body insulin sensitivity by accounting for both hepatic glucose production and peripheral glucose uptake.

Diet plays a crucial role in postprandial glucose regulation. The incretin hormones, glucose-dependent insulinotropic polypeptide (GIP) and glucagon-like peptide-1 (GLP-1), enhance insulin secretion in response to carbohydrate intake [13,14]. GIP is primarily secreted by enteroendocrine K-cells in the duodenum and jejunum, while GLP-1 is released from L-cells in the ileum [13]. These hormones bind to receptors on pancreatic β-cells, amplifying insulin secretion in a glucose-dependent manner [13,15]. In healthy individuals, incretin hormones contribute to 50–70% of postprandial insulin secretion [16]. However, individuals with T2DM often exhibit a diminished incretin response, contributing to hyperglycemia [14,17], though the exact mechanisms remain unclear.

The rising prevalence of T2DM is particularly concerning in populations where rice is a dietary staple. A meta-analysis by Hu et al. [18] found that high white rice (WR) consumption was associated with an increased risk of T2DM, particularly in Asian populations. WR, commonly known as polished rice, consists primarily of starch and significantly contributes to dietary glycemic load [19]. Parboiling rice is a pre-milling process in which paddy rice is soaked in hot water, steamed, and dried before milling. This process reduces the glycemic index by inducing starch gelatinization and retrogradation [20]. As a result, parboiled rice (PBR) has a lower glycemic index and glycemic load due to its higher fiber content and increased resistant starch [21].

A previous study by Hamad et al. [22] demonstrated that PBR helped reduce postprandial blood glucose spikes compared to WR in both healthy individuals and those with T2D. However, the underlying mechanisms were not explored, as insulin and incretin hormone responses were not assessed.

The current study aimed to determine whether hyperglycemia following rice consumption results from insulin deficiency or insulin resistance. Specifically, it investigates whether PBR enhances insulin sensitivity by improving β-cell function, increasing glucose uptake by peripheral tissues, or stimulating GLP-1 secretion in individuals with and without T2D.

A secondary objective was to assess whether venous and capillary blood glucose concentrations differ between healthy individuals and those with T2DM and whether rice type influences these glucose homeostasis parameters.

## 2. Materials and Methods

### 2.1. Study Design and Test Rice

This study employed a crossover, single-blinded experimental design. Two types of rice were tested: traditional long-grain white rice (Daawat, LT Foods Ltd., New Delhi, India) and parboiled rice (Uncle Ben’s, Mars, Inc., Houston, TX, USA), both purchased from a local market. Participants consumed either parboiled rice or white rice, ensuring an equal amount of available carbohydrates, following an overnight fast of 8 to 10 h, with a washout period of at least one week between test days. The rice was consumed within 10–12 min, along with 250 mL of water to facilitate swallowing. Physical activity was restricted during the 120 min postprandial period.

Rice samples were prepared in accordance with international guidelines for food safety and handling [23]. A weighed amount of rice, corresponding to 50 g of available carbohydrates (total carbohydrates minus dietary fiber), was used for testing. This was equivalent to 62 g of uncooked white rice cooked with 120 mL of water and 64 g of uncooked parboiled rice cooked with 210 mL of water. The rice was prepared using a rice cooker (Westinghouse Rice Cooker WST3007 ZE, Columbia, MO, USA), purchased from a local market, following the manufacturer’s cooking instructions. To enhance palatability, each serving included 2.5 g of butter and ½ teaspoon of salt. The study was approved by the Ethics Committee of the Health Science Center, Kuwait, Reference #: VDR/EC 13454, dated 18 April 2018, and the Ministry of Health, Kuwait, Reference #: MOH 4090, dated 12 July 2018. Written informed consent was obtained from all participants.

### 2.2. Recruitment of Subjects

Participants who met the eligibility criteria were recruited from Ahmadi Hospital, Kuwait. The sample size was determined based on prior blood GLP-1 measurements, with an α-error of 0.05 and a β-error of 0.20, resulting in a required minimum of seven subjects per group [24].

Inclusion and exclusion criteria:

- Healthy group: Participants aged ≥ 21 years with a body mass index (BMI) within the normal range (18.5–24.9 kg/m^2^) were included. Exclusion criteria included pregnancy, chronic disorders, use of hypoglycemic agents, smoking, and participation in high-intensity athletic activities.
- T2DM group: Participants aged ≥ 21 years with stable renal function for at least six months and a stable dose of oral hypoglycemic agents for at least three months were included. Exclusion criteria included pregnancy, end-stage diabetes complications, multiple insulin dosages, recent T2DM diagnosis, and the use of GLP-1-based oral hypoglycemic medications (specifically DPP-IV inhibitors, such as sitagliptin, saxagliptin, linagliptin, and others, which prolong endogenous GLP-1 activity by preventing its degradation).

### 2.3. Measurements

Anthropometric and physiological measurements, including height, weight, blood pressure, and HbA1c, were recorded for all participants. Weight and height were measured using a scale with 0.1 kg accuracy (SECA 284, GmbH & Co., Hamburg, Germany) and a stadiometer (Seca) with 0.1 cm accuracy, respectively. Brachial blood pressure (BP) was measured in duplicate using a Dinamap V100 BP monitor (Buckinghamshire, United Kingdom). Venous catheters were placed by a phlebotomist for blood sample collection from the forearm using specific vacutainers (Becton-Dickinson, B-D, Franklin Lakes, NJ, USA).

Serum insulin and plasma total GLP-1 concentrations were measured at fasting and at 15, 30, 60, and 120 min postprandially. Venous blood glucose was assessed at the same time points, with additional measurements at 45 and 90 min. Capillary blood glucose was measured via finger-prick using a handheld glucometer (OneTouch Ultra, LifeScan, Inc., Milpitas, CA, USA) and a Monojector Lancet Device (Covidien, Dublin, Ireland) at fasting (time 0) and at 15, 30, 45, 60, 90, and 120 min postprandially, following previously reported procedures [22].

### 2.4. Sample Analysis

At all time points, blood samples were kept on ice and centrifuged immediately. The separated serum was aliquoted into three 1.5 mL tubes and stored at −80 °C until analysis. For GLP-1 analysis, blood samples were collected in BD P800 Blood Collection Tubes (Becton-Dickinson, New Jersey, USA), containing a dipeptidyl peptidase-4 inhibitor to prevent GLP-1 degradation.

- Insulin analysis: Performed using the Diametra Insulin ELISA kit (DCM076-8-Ed 09/2018, REF DKO076).
- GLP-1 analysis: Conducted in duplicate using the GLP-1 Total ELISA kit (96-well plate assay, Cat.# EZGLP1T-36K, EZGLP1T-36BK).
- Plasma glucose: Assessed using the Beckman Coulter Oxygen Electrode, a SYNCHRON system in the biochemical analytical lab of the Kuwait Ministry of Health. A certified technician, blinded to participant identities, conducted the analysis.
- Insulin resistance (HOMA-IR) and β-cell function (HOMA-B) were calculated using the homeostatic model assessment (HOMA):
  ◦ HOMA-IR = (fasting plasma insulin × fasting plasma glucose)/22.5 [7]
  ◦ HOMA-B = (20 × fasting plasma insulin)/(fasting plasma glucose − 3.5) [9]
- Matsuda Index (MI), which assesses insulin sensitivity during an oral glucose tolerance test (OGTT), was calculated using glucose and insulin data at 0, 30, 60, and 120 min:
  ◦ MI = 10,000/√(fasting glucose × fasting insulin × mean glucose × mean insulin) [12].
- The Disposition Index (DI), which evaluates β-cell compensation for insulin resistance, was computed as:
  ◦ DI = [(postprandial insulin − basal insulin)/(postprandial glucose − basal glucose) × 18] × MI [10].
- Body Mass Index (BMI) was calculated as:
  ◦ BMI = weight (kg)/height (m^2^)
- HbA1c was measured using a Tosoh Automated Glycohemoglobin Analyzer HLC-723G8.
- Incremental areas under the curve (AUC) for glucose, insulin, and GLP-1 were calculated using the trapezoidal rule, excluding areas below baseline [25].

### 2.5. Statistical Analysis

Statistical analyses were performed using SPSS v27 and GraphPad Prism 9. Data were analyzed using analysis of variance (ANOVA) to assess the effects of time and treatment (rice type) on postprandial blood glucose, insulin, and GLP-1 concentrations in both healthy individuals and those with T2DM.

Due to unequal sample sizes and variances between groups, non-parametric tests were applied. When comparisons involved more than two groups (e.g., HOMA-B, DI, MI, AUCs for insulin, and GLP-1), the Kruskal–Wallis test of significance was used. Non-parametric tests were used for comparing fasting glucose, insulin, and HOMA-IR between the two groups before rice consumption. Also, postprandial glucose responses and AUCs (0–120 min) were compared between rice types using the Mann–Whitney U test. For comparisons within the healthy group, independent samples *t*-tests were performed, and effect sizes were calculated to assess differences in mean responses between rice types. Statistical significance was set at *p* ≤ 0.05.

## 3. Results

### 3.1. Subjects

**Figure 1:**
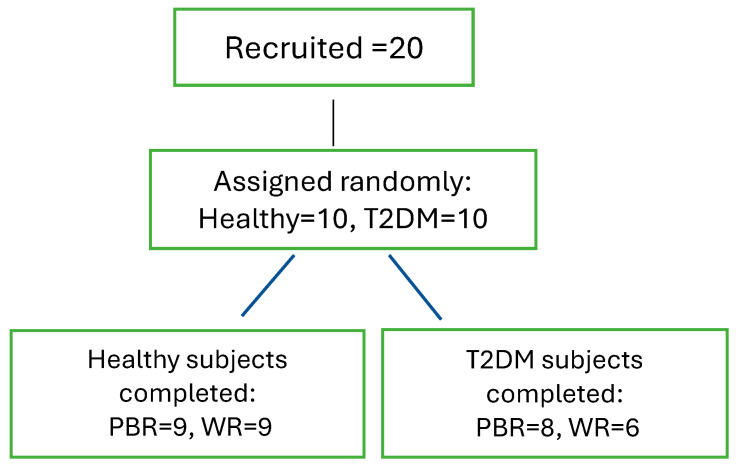
Subjects’ Recruitment and Completion of the Study. Note: The COVID-19 pandemic occurred during the study, therefore, all sessions could not be completed.

Among the total 20 subjects (n = 10 per group) recruited, 9 healthy participants completed both sessions, parboiled rice (PBH) and white rice (WRH), while in the T2DM group, 8 participants completed the PBR (PBD) session, and 6 completed the WR (WRD) session. Detailed information is provided in Figure 1.

### 3.2. Demographic Characteristics

The demographic characteristics of the study groups are summarized in Table 1. Healthy volunteers were younger and had BMI values within the normal range, whereas participants with T2DM were older and had BMI values in the overweight to obese range. As expected, HbA1c levels differed between the groups, while blood pressure remained within the normal range for both.

### 3.3. Biochemical Parameters Between the Two Groups After Consumption of the Test Rice

#### 3.3.1. Fasting Values

**Figure 2:**
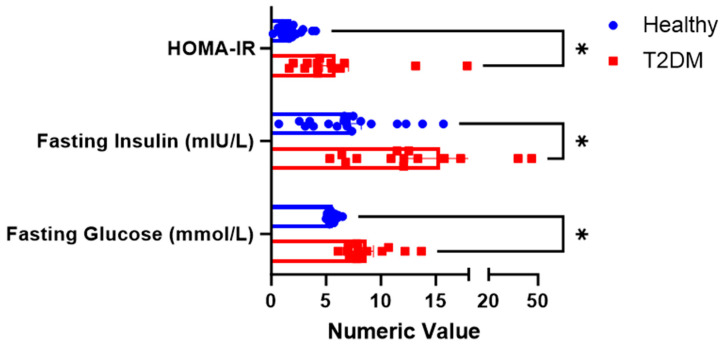
Fasting physiologic parameters between the two groups. * The superscript asterisk suggests a significant difference between the groups: HOMA_IR, and Fasting Glucose at *p* < 0.00; Fasting Insulin at *p* = 0.008.

Fasting glucose and fasting insulin were significantly higher among the subjects with T2DM than in healthy volunteers. The HOMA-IR calculated from the fasting glucose and insulin produced was also significantly higher in T2DM, as shown in Figure 2.

#### 3.3.2. Postprandial Glucose Responses

##### Blood Glucose Concentrations After White Rice (WR) and Parboiled Rice (PBR): Capillary and Venous Methods

The mean fasting capillary blood glucose concentration was significantly lower in healthy participants (4.91 ± 0.41 mmol/L) compared to those with T2DM (9.01 ± 1.75 mmol/L). In healthy individuals, glucose levels peaked at 30 min postprandially and declined steadily over the 120-min period. No significant differences in glucose concentrations were observed between WR and PBR at 15 and 30 min during the absorptive phase. However, during the disposal phase, when glucose is cleared by peripheral tissues, PBR resulted in significantly lower glucose concentrations at 60, 90, and 120 min in both groups, indicating faster glucose clearance compared to WRline 299.

**Figure 3:**
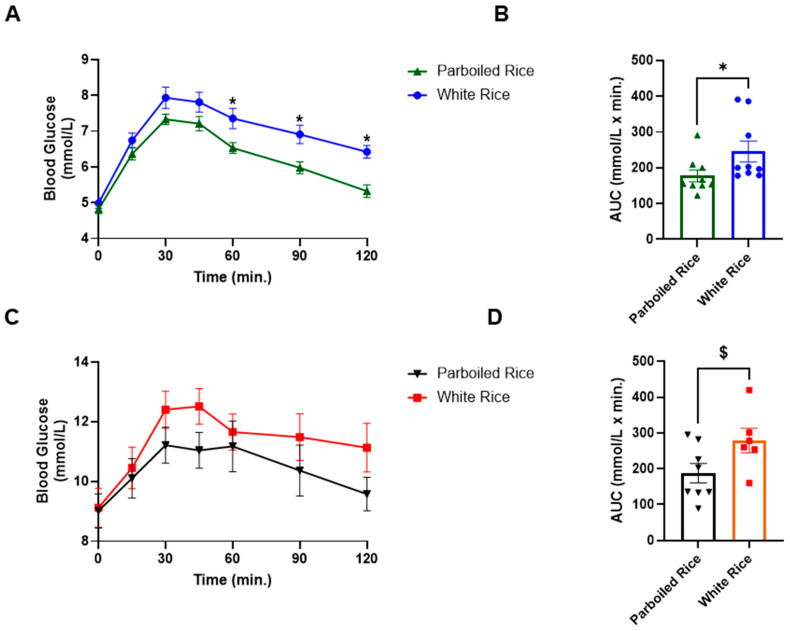
Capillary blood glucose response curves and AUC in healthy subjects and subjects with T2DM to parboiled rice and white rice. Key: (**A**,**B**) = Healthy subjects; (**C**,**D**) = People with T2DM. * Represents significant difference at *p* < 0.05, $ represents a trend in significance at *p* = 0.051.

In healthy participants, PBR led to glucose reductions of 0.6, 0.6, 0.82, 0.82, and 0.92 mmol/L at 30, 45, 60, 90, and 120 min, respectively, relative to WR. These differences were statistically significant at 60, 90, and 120 min (*p* < 0.05), with large effect sizes (η^2^ = 0.679, 0.658, and 0.535, respectively). The AUC for glucose following PBR (177.86 ± 50.02 mmol·min/L) was significantly lower than that for WR (245.56 ± 87.78 mmol·min/L, *p* = 0.047; η^2^ = 71.438), indicating a reduced overall glycemic response (Figure 3).

Among participants with T2DM, a similar pattern was observed. PBR resulted in greater reductions in glucose concentrations compared to WR, such as 1.19, 1.48, 0.48, 1.12, and 1.55 mmol/L at 30, 45, 60, 90, and 120 min, respectively. However, these differences did not reach statistical significance at any time point (*p* > 0.05). The AUC for PBR (187.38 ± 75.87 mmol·min/L) was lower than for WR (278.83 ± 84.28 mmol·min/L), with a trend toward significance (*p* = 0.051) (Figure 3).

**Figure 4:**
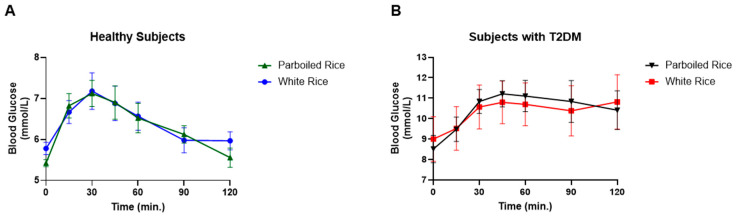
Venous blood glucose response in healthy subjects and subjects with T2DM to parboiled rice and white rice. (**A**) Healthy subjects, (**B**) Subject with T2DM.

Fasting venous glucose levels also differed significantly between groups. In healthy participants, glucose peaked at 30 min, whereas in those with T2DM, the peak occurred at 45 min. No significant differences in venous glucose concentrations were observed between WR and PBR at any time point in either group. However, glucose response patterns diverged. In healthy participants, venous glucose levels returned to baseline by 120 min for both rice types, while in T2DM participants, glucose levels remained elevated throughout the 120 min period (Figure 4). The AUCs for venous glucose did not differ significantly between rice types in either group.

#### 3.3.3. Insulin Response

Fasting insulin levels differed significantly between the two study groups, with T2DM participants exhibiting nearly twice the levels observed in healthy subjects (*p* = 0.01). However, postprandial insulin responses did not differ significantly between the rice types or between the groups overall.

In healthy individuals, insulin concentrations peaked at 15 min following ingestion of both rice types and gradually returned to baseline over 120 min. The decline in insulin levels was more pronounced after PBR compared to WR, although the difference was not statistically significant. In contrast, T2DM participants showed a delayed insulin peak at 30 min, and insulin concentrations remained elevated throughout the 120 min period. While insulin levels were slightly higher after PBR than WR, the differences were not statistically significant.

**Figure 5:**
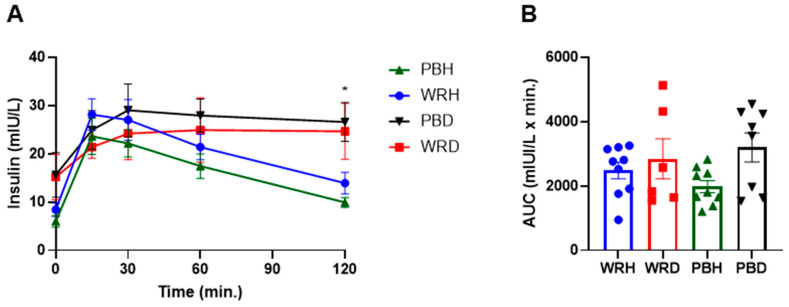
Changes in postprandial insulin response after consumption of parboiled rice and white rice. Each point represents mean ± SEM. * Insulin concentration significantly different between PBH and PBD at 120 min at *p* < 0.05. Note: PBH = parboiled rice in healthy subjects; WRH = white rice in healthy subjects; PBD = parboiled rice in diabetics; WRD= white rice in diabetics. (**A**) Insuline response, (**B**) Insulin AUC.

At 120 min, insulin concentrations differed significantly between healthy and T2DM subjects. In particular, insulin levels after PBR in healthy participants (PBH) were significantly lower than those in the T2DM group after both PBD and WRD, with adjusted *p*-values of 0.002 and 0.033, respectively. Additionally, insulin levels after WR in healthy participants (WRH) were significantly lower than those after PBD (unadjusted *p* = 0.031), though there was no significant difference between WRH and WRD at 120 min (Figure 5).

There were no significant differences in the insulin area under the curve (AUC) between rice types within either study group. However, the unadjusted insulin AUC for PBH was significantly lower than that for PBD (*p* = 0.049). This difference was not statistically significant after applying the Bonferroni correction.

#### 3.3.4. GLP-1 Responses

No significant differences in GLP-1 responses to PBR or WR were observed within either subject group. However, the AUC for GLP-1 concentrations was approximately twice as high in healthy participants compared to those with T2DM following both rice types. Specifically, after WR, the AUCs were 1002 ± 428 mmol·min/L in healthy participants (WRH) and 507 ± 150 mmol·min/L in those with T2DM (WRD). After PBR, the AUCs were 1515 ± 697 mmol·min/L in PBH and 804 ± 501 mmol·min/L in PBD. A statistically significant difference was observed only between PBH and WRD, with GLP-1 levels being significantly higher in PBH compared to T2DM participants after WR (Adjusted *p* = 0.015) (Figure 5).

#### 3.3.5. HOMA-IR, Matsuda Index, HOMA-B, and Disposition Index

**Figure 6:**
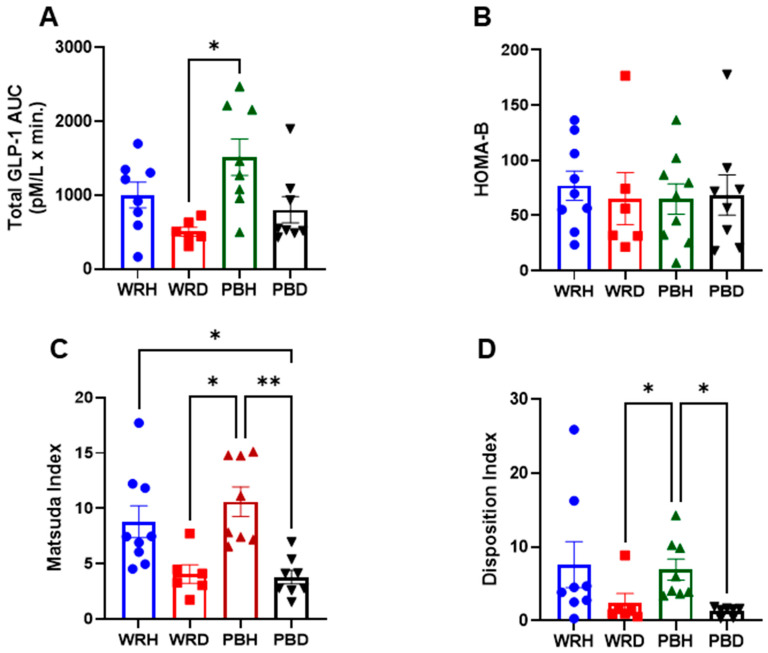
Total GLP-1 AUC, HOMAR-B, Matsuda Index, and Disposition Index generated from the responses to either WR or PBR in healthy subjects or those with T2DM. Data are presented as AUC Mean ± SEM; The results are significant at * *p* < 0.05, and ** *p* < 0.01. Note: PBH = parboiled rice in healthy subjects; WRH = white rice in healthy subjects; PBD = parboiled rice in diabetics; WRD= white rice in diabetics. (**A**) Glp-1, (**B**) HOMA-B, (**C**) Matsuda Index, (**D**) Disposition Index.

Insulin resistance, as measured by the homeostatic model assessment for insulin resistance (HOMA-IR), was significantly higher in T2DM participants compared to healthy individuals (*p* < 0.05; Figure 1). Since HOMA-IR is calculated from fasting insulin and glucose levels, it effectively reflects insulin resistance differences between diabetic and non-diabetic states independent of postprandial influences. HOMA-B, which also derives from fasting values and estimates β-cell function, did not differ significantly between the groups (Figure 6).

The Matsuda Index (MI), which incorporates both fasting and postprandial glucose and insulin values to assess whole-body insulin sensitivity, was significantly higher after PBR in healthy participants compared to both PBR and WR in T2DM participants. MI after PBH and WRH was significantly greater than after PBD (unadjusted *p* = 0.000 and *p* = 0.006), respectively. Similarly, both PBH and WRH were significantly higher than WRD (unadjusted *p* = 0.002 and *p* = 0.027), respectively. After Bonferroni correction, the significance of these comparisons slightly diminished: PBH vs. PBD (adjusted *p* = 0.001), PBH vs. WRD (adjusted *p* = 0.012), and WRH vs. PBD (adjusted *p* = 0.038).

The Disposition Index (DI), which accounts for insulin secretion relative to insulin sensitivity, was also significantly higher in PBH compared to PBD and WRD (adjusted *p* = 0.009 and *p* = 0.037, respectively). DI after WRH was higher than PBD and WRD (unadjusted *p* = 0.018 and 0.049), though these differences were not significant after Bonferroni correction. No significant differences in DI were observed between rice types within either group (Figure 6).

## 4. Discussion

Insulin resistance, often presenting with hyperglycemia and hyperinsulinemia, is a well-established risk factor for type 2 diabetes mellitus [26]. In this study, participants with T2DM exhibited significantly higher fasting glucose despite oral hypoglycemic therapy. This raised questions about whether hyperglycemia stemmed from β-cell dysfunction, insulin resistance, or both. We also evaluated whether PBR could attenuate postprandial hyperglycemia by enhancing insulin secretion or peripheral glucose disposal, compared with WR. Furthermore, comparisons of glycemic responses to WR and PBR were made using both venous and capillary blood glucose measurements in healthy and T2DM subjects.

Handheld glucometers, commonly used for at-home monitoring, provide capillary glucose values, yet limited studies directly compare these with venous measurements. In our study, fasting glucose values from both measurement methods confirmed expected differences between groups. Postprandially, T2DM subjects exhibited a delayed glucose peak compared to healthy individuals, regardless of the sampling method.

Notably, capillary glucose following PBR intake was lower than WR at multiple time points in both groups, reaching statistical significance at 60, 90, and 120 min in healthy individuals. This resulted in a significantly lower AUC. In T2DM subjects, although the capillary glucose decrease after PBR was more pronounced, it did not reach statistical significance, likely due to greater variability and the limited sample size, which was designed for GLP-1 assessment rather than glucose measurement [24]. A previous study from our lab with a larger sample size found significant reductions in glucose at each time point in healthy individuals and between 60 and 120 min in those with T2DM [22]. Despite the lack of significance in this pilot, PBR led to a 36% reduction in glucose AUC in T2DM subjects versus a 28% reduction in healthy participants, demonstrating a biologically meaningful reduction. These findings are consistent with previous studies reporting 30–38% reductions in glucose AUC with PBR consumption [22,27].

In contrast, venous glucose measurements did not show significant differences between PBR and WR in either group. Healthy individuals returned to baseline more rapidly, while glucose levels remained elevated in T2DM participants throughout the study. These findings emphasize how physiological status (diabetic vs. non-diabetic) and the measurement method (capillary vs. venous) can influence glycemic response assessments. While PBR improved capillary glucose clearance in both groups, its effects were less apparent in venous samples.

Prior studies indicate that arterial and capillary glucose levels are approximately 7% higher than venous glucose in healthy individuals [28,29], and similar trends are observed in both T1DM and T2DM patients [30,31,32], likely due to glucose diffusion into tissues before returning to the veins [33]. Given that WHO uses venous plasma glucose for diabetes diagnosis [34], whereas FAO and WHO accept both capillary and venous glucose for glycemic index testing [35], our results underscore the importance of selecting appropriate measurement methods when assessing insulin sensitivity and glycemic responses.

The delayed postprandial glucose peak in T2DM subjects (at 45 min) versus healthy individuals (30 min) was mirrored by insulin responses. In healthy subjects, insulin returned to baseline by 120 min, while it remained elevated in T2DM subjects. Insulin AUC over 120 min was lower after PBR than WR in healthy individuals (~500 mU/L·min difference), but higher in T2DM subjects (~360 mU/L·min difference). PBR elicited a greater insulin response in T2DM subjects (3199 ± 1273 mU/L·min) than in healthy controls (1980 ± 568 mU/L·min), suggesting a compensatory insulin secretion in response to hyperglycemia. Although not statistically significant, these trends suggest PBR may support insulin secretion in T2DM.

HOMA-B values did not differ significantly between groups, indicating preserved β-cell function in T2DM subjects. This could reflect residual β-cell capacity or medication-induced improvement. Previous studies support the notion that T2DM patients may exhibit normal HOMA-B values due to intact or pharmacologically supported insulin production [36]. However, despite normal fasting insulin levels, these individuals often exhibit inadequate insulin action, pointing to peripheral insulin resistance [37,38]. In this study, T2DM subjects exhibited elevated fasting glucose and insulin, yielding high HOMA-IR values, highlighting the role of insulin resistance in persistent hyperglycemia despite adequate insulin secretion.

The Disposition Index (DI), which combines insulin secretion and sensitivity, offers a more nuanced assessment of β-cell function. Lower DI values in T2DM participants indicated an inadequate β-cell response to insulin resistance. PBR consumption had led to significantly higher DI values compared to WR, but only in healthy individuals. These lower values of DI, and underscore the inability of β-cells to adequately compensate for decreased insulin sensitivity, highlighting the progressive nature of T2DM. Although the PBR consumption had increased the secretion of insulin in people with T2DM, it did not reach statistical significance, potentially due to the small sample size and the acute nature of this study. A longer-term study with a low-carbohydrate diet showed substantial increases in DI and C-peptide response in T2DM individuals [39], underscoring the therapeutic potential of dietary interventions.

HOMA-IR was significantly higher in T2DM compared to healthy subjects (5.4 vs. 1.81, *p* < 0.001), confirming systemic insulin resistance [26,40,41]. Meanwhile, the Matsuda Index (MI), reflecting peripheral insulin sensitivity, was significantly lower in T2DM subjects, supporting impaired glucose uptake. MI values below 4.3 predict insulin resistance [26] and correlate with euglycemic clamp results [10,42]. In contrast, MI in healthy subjects doubled after WR and tripled after PBR, suggesting enhanced insulin sensitivity. Notably, less insulin was required to clear glucose after PBR compared to WR, not only in healthy but also in T2DM subjects, highlighting its favorable metabolic profile. Our results are corroborated by others who also demonstrated the consumption of PBR’s association with enhanced insulin sensitivity relative to WR [27,43].

The role of GLP-1 in mediating these effects is crucial. GLP-1 enhances glucose-stimulated insulin secretion, but its levels are often impaired in T2DM [44,45,46,47,48,49]. A recent systematic review and meta-analysis of studies comparing GLP-1 between people with and without diabetes found slight and inconsistent differences [50,51]. Our study showed a two-fold higher GLP-1 AUC in healthy vs. T2DM individuals after WR, and increased GLP-1 levels in both groups after PBR (~500 pM·min increase in healthy and ~300 pM·min in T2DM). Research on the effects of parboiled rice consumption on GLP-1 secretion in humans is scarce. Our study is the first to assess the PBR on GLP-1 secretion in both healthy individuals and people with T2DM.

The increased GLP-1 secretion observed following PBR intake may be attributed, in part, to the formation of resistant starch (RS). During parboiling, hydrothermal treatment gelatinizes the starch granules, and subsequent cooling promotes retrogradation, a process that converts gelatinized starch into RS, a form that resists enzymatic digestion in the small intestine and functions similarly to dietary fiber [52,53]. This transformation results in a denser, more compact starch matrix that limits the accessibility of α-amylase and other digestive enzymes [54,55]. The formation of amylose-lipid complexes during parboiling further reduces enzymatic digestibility [43]. Notably, retrograded starch remains resistant even after reheating, retaining its functional properties [27]. These structural changes collectively lower the glycemic response and may enhance GLP-1 secretion.

Compared to white rice, PBR contains a lower proportion of rapidly digestible starch and a higher proportion of slowly digestible starch and RS, factors associated with a more gradual glucose release and improved glycemic control [53,56]. These structural modifications also alter the rice’s physical properties, such as swelling power, solubility, and pasting characteristics, all of which influence starch digestibility and digestion kinetics [57]. In the present study, this slower digestibility is reflected in the delayed and attenuated postprandial glucose and insulin peaks following PBR consumption. These effects were evident in both healthy and T2DM participants, highlighting the potential metabolic benefits of incorporating PBR as a staple carbohydrate source in populations at risk for impaired glucose homeostasis.

Although not statistically significant across all comparisons, the increased GLP-1 responses observed after PBR intake may also be mediated by colonic fermentation of RS. RS serves as a substrate for gut microbiota, leading to the production of short-chain fatty acids (SCFAs), including acetate, propionate, and butyrate. These SCFAs have been shown to stimulate GLP-1 secretion via activation of G-protein coupled receptors such as FFAR2 (GPR43), which are expressed on enteroendocrine L cells [58,59,60,61]. Incretin hormones like GLP-1 enhance glucose-dependent insulin secretion and may contribute to the improved postprandial insulin response and glycemic regulation observed with PBR.

In summary, the structural characteristics of parboiled rice, arising from starch gelatinization, retrogradation, and recrystallization, appear to modulate starch digestibility and promote favorable metabolic responses. PBR thus emerges as a promising dietary alternative to white rice, with evidence suggesting that it can enhance insulin secretion, facilitate glucose uptake, and attenuate postprandial glycemic excursions. These effects may be partially mediated by PBR’s capacity to stimulate intestinal GLP-1 secretion, a key incretin hormone that plays a central role in postprandial glucose regulation. Given that impaired GLP-1 secretion is closely associated with the pathophysiology of type 2 diabetes mellitus (T2DM), regular dietary incorporation of PBR may offer dual benefits: improving insulin sensitivity and enhancing endogenous incretin responses. Nonetheless, further research is needed to elucidate the precise mechanistic pathways involved and to assess the influence of inter-individual variability, including genetic factors, on these outcomes.

## 5. Conclusions

Participants with T2DM exhibited higher fasting glucose, increased HOMA-IR, lower Matsuda Index, and reduced Disposition Index compared to healthy individuals. Consumption of PBR significantly improved postprandial glycemia in healthy participants and showed modest benefits in T2DM subjects. These effects were accompanied by enhanced insulin sensitivity and increased GLP-1 secretion. Mechanistically, the glycemic benefits of PBR may be attributed to its altered starch structure, specifically, increased RS content resulting from parboiling-induced gelatinization and retrogradation, which reduces starch digestibility and slows glucose absorption. The observed increase in GLP-1 further suggests that PBR may promote gut hormone responses conducive to improved glycemic control. Together, these findings highlight PBR as a functional dietary strategy that may aid in managing insulin resistance and mitigating T2DM risk. Further long-term studies are needed to validate these effects in larger and more diverse populations. Furthermore, capillary glucose measurements were more sensitive to dietary intervention than venous glucose.

## 6. Strengths and Limitations

This study has several strengths. First, it employed a crossover design, which is particularly effective in controlling for individual variability among participants. Second, it included comprehensive metabolic assessments, encompassing insulin secretion, β-cell function, the Matsuda Index, the Disposition Index, and GLP-1 secretion, providing a thorough evaluation of glycemic and hormonal responses. Third, the study compared capillary glucose concentrations with venous glucose concentrations, offering a more nuanced understanding of glucose dynamics.

However, there are also limitations to consider. The sample size was relatively small, based on power calculations for GLP-1 assessment, and was further reduced due to challenges during the COVID-19 pandemic. Many initially recruited participants were unable to complete the study, as healthcare facilities were largely closed except for urgent care, leading to the premature termination of data collection. Additionally, this was an acute feeding pilot study; therefore, long-term studies are needed to confirm and expand upon these findings.

## Figures and Tables

**Table 1:** Demographic and Clinical Characteristics of Study Groups.

Variable	Diabetic (*n* = 8)	Healthy (*n* = 9)
Gender		
Male/Female (*n*)	3/5	4/5
Age (years) Mean ± SD	45.96 ± 11.34	32.9 ± 2.64 *
BMI (kg/m^2^) Mean ± SD	31.23 ± 4.50	23.54 ± 0.74 *
Blood Pressure (mm Hg) Mean ± SD		
Systolic	117.33 ±11.82	112.5 ± 9.99
Diastolic	79.33 ± 7.07	80 ± 3.53
HbA1c (%)	6.75 ± 0.67	4.96 ± 0.28 *

* Significantly different at *p* < 0.05.

## Data Availability

The original contributions presented in this study are included in the article. Further inquiries can be directed to the corresponding author.
